# Vegan diet as a possible cause of mental and physical disorders due to vitamin B12 deficiency in an adolescent patient

**DOI:** 10.1192/j.eurpsy.2022.1100

**Published:** 2022-09-01

**Authors:** E. Gruber, S. Martic Biocina

**Affiliations:** 1 Pscyhiatri hospital Sct.Hans, Department Of Forensic Psychiatry, Roskilde, Denmark; 2 University Psychiatric Hospital Vrapce, Department Of Social Psychiatry, Zagreb, Croatia

**Keywords:** adolescent, vegan diet, deficiency vitamin B12, Mental Disorders

## Abstract

**Introduction:**

Recent studies show that a vegan diet causes a deficiency of vitamins (especially B12) and minerals. This can lead to severe physical and mental illnesses. On the other hand, the vegan diet is recommended as a preventative measure against cardiovascular diseases and is a growing trend among young people in developed countries for ideological reasons such as animal welfare and climate protection.

**Objectives:**

To show the importance of anamnesis of nutrition and vitamin B12 status in treatment of varied mental and physical symptoms in an adolescent.

**Methods:**

The poster shows the case study of an adolescent girl who sought psychological help for nightmares and symptoms of anxiety and depression, as well as physical symptoms in the form of disturbed menstruation, fatigue and weakness, lethargy, dizziness, undifferentiated abdominal pain with nausea, and weight gain. All of which affected her academic success at university and daily functioning. An anamnesis showed that she has been following a vegetarian diet for 4 years and a vegan diet for two months.

**Results:**

Laboratory tests showed a deficiency of vitamin B12 (130 pmol/L) and 25-Hydroxy-Vitamin D(D3+D2) (47 nmol/l) and slightly elevated TSH levels (4,2x10-3 IU/L). These tests can explain the patient’s symptoms. Other laboratory results were in the normal range. A treatment with psychological therapy and vitamin supplements was commenced. Discussion reviews, among else, recent literature findings on correlation of vitamin B12 deficiency and a vegan diet.

**Conclusions:**

Nutrition and vitamin B12 status should be investigated during anamnesis of adolescent patients presenting with varied mental and physical symptoms.

**Disclosure:**

No significant relationships.

